# Comparative Proteomic Profiling of Blood Plasma Revealed Marker Proteins Involved in Temporal Lobe Epilepsy

**DOI:** 10.3390/ijms25147935

**Published:** 2024-07-20

**Authors:** Yury E. Glazyrin, Dmitry V. Veprintsev, Elena E. Timechko, Zoran Minic, Tatiana N. Zamay, Diana V. Dmitrenko, Maxim V. Berezovski, Anna S. Kichkailo

**Affiliations:** 1 Laboratory for Digital Controlled Drugs and Theranostics, Federal Research Center “Krasnoyarsk Science Center of the Siberian Branch of the Russian Academy of Science”, Akademgorodok 50, 660036 Krasnoyarsk, Russia; d_veprintsev@mail.ru (D.V.V.); tzamay@yandex.ru (T.N.Z.); annazamay@yandex.ru (A.S.K.); 2 Laboratory for Biomolecular and Medical Technologies, Prof. V.F. Voino-Yasenetsky Krasnoyarsk State Medical University, Partizana Zheleznyaka 1, 660022 Krasnoyarsk, Russia; 3 Department of Medical Genetics and Clinical Neurophysiology, Prof. V.F. Voino-Yasenetsky Krasnoyarsk State Medical University, Partizana Zheleznyaka 1, 660022 Krasnoyarsk, Russia; e.e.timechko@yandex.ru (E.E.T.); mart2802@yandex.ru (D.V.D.); 4 Department of Chemistry and Biomolecular Sciences, University of Ottawa, 10 Marie-Curie, Ottawa, ON K1N 6N5, Canada; zminic@uottawa.ca (Z.M.); maxim.berezovski@uottawa.ca (M.V.B.)

**Keywords:** biomarkers, temporal lobe epilepsy, proteomics, mass spectrometry

## Abstract

Temporal lobe epilepsy has various origins, involving or not involving structural changes in brain tissue. The mechanisms of epileptogenesis are associated with cell regulation and signaling disruptions expressed in varied levels of proteins. The blood plasma proteomic profiling of temporal lobe epilepsy patients (including magnetic resonance imaging (MRI)-positive and MRI-negative ones) and healthy volunteers using mass spectrometry and label-free quantification revealed a list of differently expressed proteins. Several apolipoproteins (APOA1, APOD, and APOA4), serpin protease inhibitors (SERPINA3, SERPINF1, etc.), complement components (C9, C8, and C1R), and a total of 42 proteins were found to be significantly upregulated in the temporal lobe epilepsy group. A classification analysis of these proteins according to their biological functions, as well as a review of the published sources, disclosed the predominant involvement of the processes mostly affected during epilepsy such as neuroinflammation, intracellular signaling, lipid metabolism, and oxidative stress. The presence of several proteins related to the corresponding compensatory mechanisms has been noted. After further validation, the newly identified temporal lobe epilepsy biomarker candidates may be used as epilepsy diagnostic tools, in addition to other less specific methods such as electroencephalography or MRI.

## 1. Introduction

Epilepsy affects more than 70 million people and is one of the most common neurological disorders in the world [1]. Temporal lobe epilepsy (TLE) is often accompanied by histological changes in the brain tissue, such as hippocampal sclerosis (HS), characterized by the loss of pyramidal neurons and gliosis in the C1 and C3 regions, as well as in the areas of the Ammon’s horn [2]. HS is one of the most common causes of drug-resistant epilepsy in adults [1]. However, according to magnetic resonance imaging (MRI), some patients do not have any structural changes in the brain. Despite advances in research on the neuronal mechanisms underlying epileptogenesis and the spread of new anticonvulsants, there are no pharmacological agents for the prevention of epileptogenesis; the mechanisms of epileptogenesis do not coincide with the mechanisms of ictogenesis, the process leading to seizure [3]. Along with the lack of effective pharmacological approaches and disease-modifying therapies, the diagnosis of epilepsy also remains a clinical problem [4].

Today, diagnostics of epilepsy mostly depend on clinical examination, history, and electroencephalography (EEG) monitoring—a method that is neither specific nor sensitive [4], and also, in some cases, leads to an erroneous diagnosis.

Accordingly, there is considerable interest in the discovering and testing of new biomarkers for the earlier and more reliable diagnosis for timely treatment. Information on the specific protein expression will lead to a deeper insight into the mechanism of epileptogenesis. The proteomic profiling, characterization, and comparison of the different types of epilepsy in terms of proteins present in the blood is extremely important for the development of clinical diagnostic tools. A relevant model for the study of epilepsy is TLE, as the most common form of focal epilepsy.

Several studies evaluated the levels of the selected candidate biomarker proteins in blood or cerebrospinal fluid (CSF) [5,6]. The whole proteomic quantitative analysis offers an unbiased screening approach—and gives a general idea of the changes in protein levels [7]. Plasma proteomic studies have provided information on the potential biomarkers for several neurological disorders [8], but there are few proteomic studies of epilepsy. Biomarker discovery performed on animal and human tissue models [9] revealed the aberrant expression of markers of neurodegeneration and neuroinflammation, as well as changes in the energy metabolism systems. Only a few studies evaluated patient plasma biomarkers, where the authors found several differentially expressed proteins associated with the immune response in children with Rolandic epilepsy [10]. In a recent study [11], some proteins associated with neuroinflammation and neurodegeneration were tested in plasma as predictive markers of the course of TLE in relation to drug resistance.

The majority of TLE patients took antiepileptic drugs (AEDs) in monotherapy or polytherapy. It is quite likely that the use of AEDs can affect protein expression. It would certainly be important to identify potential protein markers of epilepsy without taking into account the influence of AEDs, but epilepsy is a disease that requires the long-term use of AEDs. Thus, the involvement in studies of large patient groups without drug therapy seems quite difficult. In this research, we compared the proteomic profiles of the blood plasma of AED-taking patients with TLE (including MRI-positive and MRI-negative ones) and healthy volunteers to search for new markers of the disease. The proteins implicated in early studies as being associated with AEDs were rejected. The proteins involved in neuroinflammation were proposed as epileptogenesis marker candidates. The different expression of the identified proteins indicates multiple disturbances in intracellular signaling, lipid metabolism, inflammation, and impaired processes of neuronal excitability.

## 2. Results

### 2.1. Manifestation of Proteomic Differences in the Groups

A mass spectrometry analysis of plasma samples was performed for 27 temporal lobe epilepsy patients and 7 volunteers. The data analysis led to obtaining quantitative information about the distribution of 192 proteins in plasma samples of 34 participants made in triplicates. Triple repetitions were averaged without significant loss, as described in Section 4.

Principal component analysis (PCA) is a way to reduce the dimensionality of the complex dataset by diminishing the least amount of information. A multi-dimensional matrix of proteins and their quantitative indicators is reduced by projecting them onto the new axes of the principal components. At the same time, the presence of the most distinctive proteins affects the relative positions of the observations in the new co-ordinates, clearly highlighting their differences.

**Figure 1:**
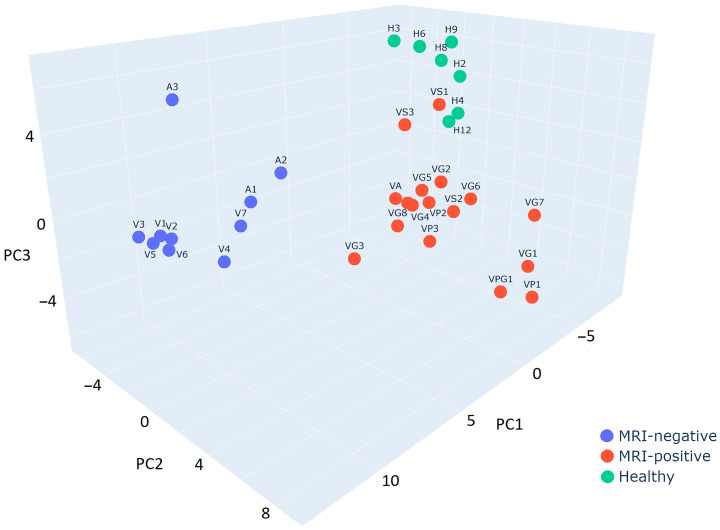
The 3D principal component analysis diagram of protein distributions for the MRI-positive epilepsy patients, MRI-negative epilepsy patients, and the healthy group. Point labels are described in the footnote to Table 1.

The manifestation of differences in proteomic profiles between MRI-negative and MRI-positive TLE groups, and also the healthy group, are illustrated in principal component co-ordinates by 3D PCA (Figure 1). The MRI-positive group is separated from the MRI-negative group, which allows us to justify the comparison of proteomic profiles of the groups to identify the most distinguishing proteins.

PCA was also used to search for proteomic differences between the groups by sex, age, and drug resistance. No distinct clusters were found, which shows that these features do not have a significant influence on the proteomic profiles of the samples.

The different arrangement of patient groups in the planes of the principal components gives grounds to compare their proteomic profiles statistically for the identification of significant protein markers of these groups. The proteomic profiles of the selected patient groups were compared in pairs, and statistically different proteins (corrected by the Benjamini–Hochberg procedure Welch’s *t*-test *p*-value < 0.01) were noted as significantly changed based on their label-free quantification (LFQ) values.

### 2.2. Proteomic Profile Comparison of the Whole Epilepsy Group (TLE) versus the Healthy Group

**Figure 2:**
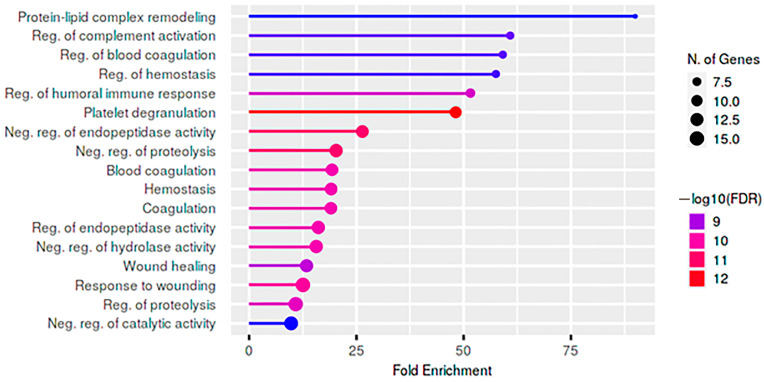
Plot of biological processes for upregulated proteins when comparing the overall epilepsy group with the healthy group.

When comparing the whole group of TLE patients with healthy controls, 42 proteins were identified as significantly upregulated in epilepsy (Table S1). An analysis of the tissue protein expression using Human Protein Atlas (HPA) demonstrated the presence of mainly liver proteins. A Gene Ontology (GO) analysis performed using ShinyGo 0.76 demonstrated the involvement of overexpressed proteins in the regulation of the following biological processes: lipid metabolism, immune response, catalytic activity, and coagulation (Figure 2).

The proteins upregulated in the epilepsy group (Table 2) are involved in the processes of the response to external stimuli, stress, and the immune response, which indicates the presence of a neuroinflammatory process that accompanies and maintains epileptogenic activity. However, the question of their specificity is still open. Proteins that regulate the process of intracellular signaling have also been identified, which demonstrates the dysregulation of the cascade of intracellular events. The altered expression of proteins that remodel the levels of plasma lipoproteins indicates a lipid metabolism disorder in patients with epilepsy.

### 2.3. Proteomic Profile Comparison of MRI-Negative and MRI-Positive Epilepsy Groups

Next, we compared the proteomic profiles of two subgroups of epilepsy, which differ on the PCA diagram—MRI-negative and MRI-positive.

The MRI-positive TLE patients are characterized by MRI-visible affected areas of the epileptogenic focus. The MRI-negative group is presented by TLE patients with recurrent unprovoked seizures, originating from the temporal lobe based on electroclinical data, in the absence of an epileptogenic lesion on a visual examination of the MRI, and without signs of hippocampal sclerosis, vascular malformations, tumors, hamartomas, etc.

**Figure 3:**
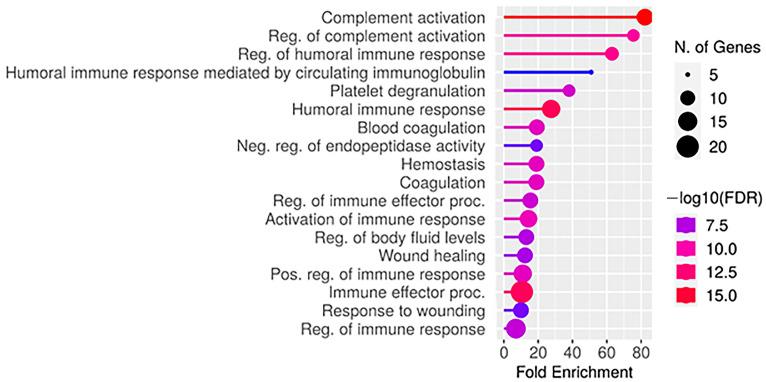
Plot of biological processes for upregulation in MRI-positive group proteins.

For these groups, a list of 46 significantly altered proteins was obtained (Table S2). Eleven proteins were found to be upregulated in the MRI-positive group. The GO analysis revealed the involvement of these proteins in the list of biological processes (Figure 3). Most of the overrepresented MRI-positive group proteins are involved in the regulation of the immune response.

**Figure 4:**
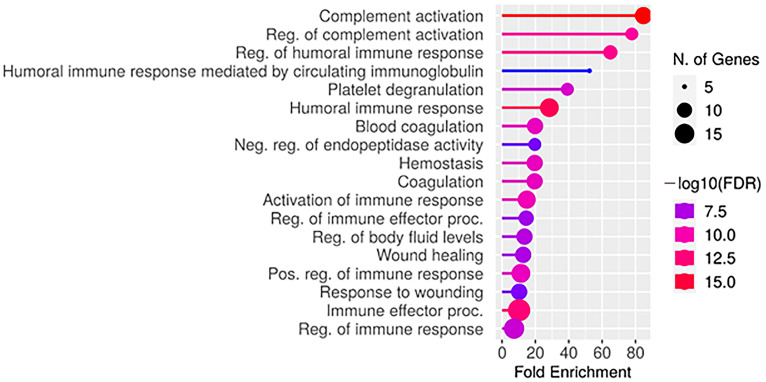
Plot of biological processes for upregulation in MRI-negative group proteins.

The group of MRI-negative patients is characterized by the upregulation of 35 proteins. The GO analysis revealed the involvement of these proteins in a number of biological processes (Figure 4).

For both groups, most of the overrepresented proteins are involved in the regulation of the immune response, which is typical for the neuroinflammatory process. Neuroinflammation is one of the leading mechanisms of epileptogenesis. However, the set of differently expressed proteins for the MRI-positive and MRI-negative groups was different. Currently, an additional examination for the MRI-negative group reveals the autoimmune nature of the disease, which explains the widespread involvement of inflammatory proteins in the pathogenesis of the disease.

Plasma proteomic profiles dependent on drug response status, age, and sex of the TLE patients were tested. No statistically significant difference was found between the drug-resistant and non-drug-resistant epilepsy groups, as well as between the two age groups (under 40 and over 40), or based on gender.

## 3. Discussion

### 3.1. Association of Plasma and Brain Proteins in Epilepsy

According to the Human Protein Atlas available online: https://www.proteinatlas.org/ (accessed on 18 July 2024), some of the proteins we discovered in plasma are detected in the brain: HPX, CP, SERPINA3, APOA1, APOD, HPR, APOC2, BCHE, SELL, AGT, VASN, IGKV4-1, ORM1, ALB, and SERPINF2. However, blood immunological inflammatory substrates have been found to play a role in the pathogenesis of many neurological disorders. In particular, some studies have demonstrated the induction of plasma inflammatory and neurotrophic markers [12]. This was also observed in animal models of temporal lobe epilepsy [13]. Moreover, a number of studies discussed the epileptogenicity of the activation of the peripheral immune system [14]. It is known that prolonged seizures are associated with an increase in the permeability of the blood–brain barrier (BBB), as well as with multiple changes in the properties of the BBB [15], which was demonstrated by the infiltration of the brain parenchyma by peripheral immune cells [16]. In addition, plasma inflammation persists long-term after an attack [17]. These data suggest the presence of a systemic response involved in epileptogenic processes, indicating that the changed plasma protein profile with epilepsy reflects changes in brain proteins.

**Figure 5:**
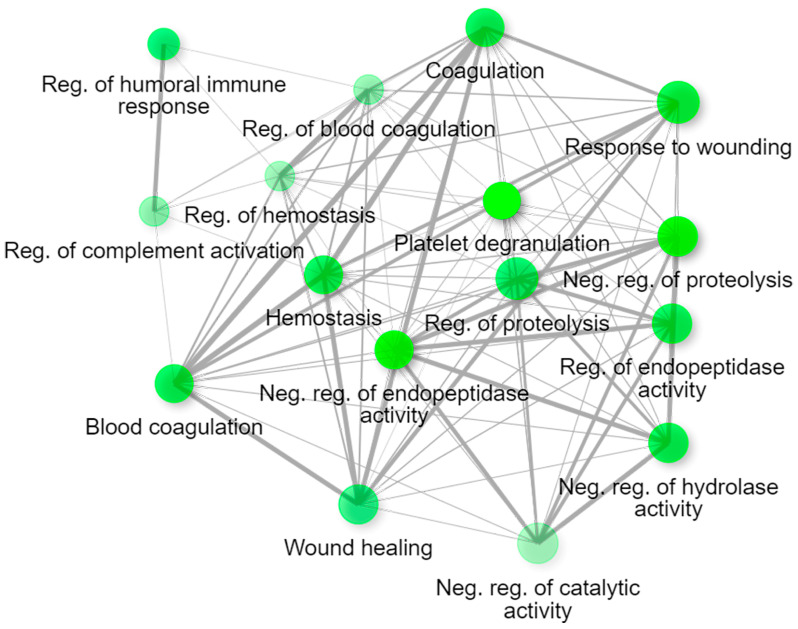
Network of biological processes of proteins with altered expression, registered by comparing plasma proteomic profiles of epilepsy patients with healthy control plasma.

The majority of the proteins with an altered expression found by the comparison of epilepsy and healthy plasma proteomic profiles are involved in immune response processes, lipid metabolism, and the regulation of peptidase activity (Figure 5). The process of epileptogenesis causes massive changes in cellular and molecular processes, as evidenced by the aberrations of proteins involved in various mechanisms. Epilepsy does not cause strictly specific dysregulations. Biochemical cascades triggered by an attack lead to changes in various biological and molecular pathways, that are not direct factors in the development of the disease.

Some of the proteins with an altered expression found in earlier studies are compiled in Table S3.

### 3.2. Structural Hippocampal Changes in Epilepsy

Hippocampal sclerosis is not considered to be a distinct disease, but likely consists of several subtypes, and the causes of this condition are currently poorly understood [2]. In addition, according to histological studies, in patients with temporal lobe epilepsy, the loss of neuronal cells and pronounced astrocytic gliosis in the hippocampus are recorded, in contrast to epilepsy not associated with sclerosis, when neurons are preserved and gliosis has no features. A decrease in synaptic proteins is a manifestation of the loss of neuronal cell bodies and dendrites, whereas an increase in glial-associated proteins is a manifestation of astrocyte proliferation and hypertrophy. It is believed to be a result of sclerosis of the hippocampus.

Traditionally, epileptogenesis is the process by which the neural network of the brain functionally changes to increase the susceptibility to epileptic seizures, which increases the likelihood of spontaneous recurrent seizures (SRSs) [18]. Therefore, the process of epileptogenesis was considered exclusively in the context of the “latent period”, i.e., as the period of time between the epileptogenic event and the onset of the first clinically detectable seizure. However, a number of studies have demonstrated that the frequency and severity of SRSs continues to increase after the first unprovoked or spontaneous attack [19,20]. Therefore, there are data that allow us to consider the process of epileptogenesis as a continuous and long-term process. It is also demonstrated that the processes of the reorganization of brain tissue, leading to the formation of the first SRS, also continue after it [18,21], which contributes to the further progression of the disease and the induction of subsequent attacks. At the same time, there are opinions that epilepsy is a progressive disease, which includes the death of neurons, gliosis, the reorganization of neural networks and the development of pharmacoresistance, which is associated directly with epileptomorphic brain activity according to the “seizures-beget-seizures” theory [22]. Therefore, the frequency and severity of attacks, as well as structural changes in the hippocampus, may be a consequence of the ongoing process of epileptogenesis.

### 3.3. Oxidative Stress Regulation

Several groups of proteins identified in our study and previously associated with epilepsy exhibit a similar pattern. Among them are apolipoproteins involved in lipid metabolism that reduce the oxidative stress caused by lipid peroxidation: APOA1, APOD, APOE, and APOA4.

APOA1 regulates the functions of neutrophils and reduces the synthesis of reactive oxygen species in brain cells. An increased content of APOA1 was found in the hippocampus tissues of mesial TLE patients [23]. Our study found APOA1 overexpression in TLE patients compared to controls, regardless of their MRI status. APOD has a high affinity for arachidonic acid (AA), which is a regulator of many inflammatory processes associated with neuronal damage [24]. APOD and its transcript are overexpressed during the acute inflammatory phase, reducing the secretion of pro-inflammatory cytokines and interferons. The early accumulation of the APOD protein in hippocampal neurons after limbic seizures has been reported [25]. A selective increase in APOD mRNA was observed in hippocampal tissues in a kainic model of epilepsy in rats [26]. It was associated with Alzheimer’s and Parkinson’s diseases [27], as well as related to oxidative stress [28], and multiple sclerosis [29]. In our study, APOD was found to be overexpressed in the plasma of TLE patients compared to controls. A high level of APOE was found to be associated with an earlier onset of temporal lobe epilepsy and was observed in TLE patients’ plasma [30]. In our study, APOE overexpression was found in the group of MRI-positive TLE patients. APOA4 is a potent inhibitor of lipid oxidation and an anti-inflammatory agent inhibiting P-selectin-mediated adhesive interactions between leukocytes and platelets. Its concentration increases in the plasma of refractory epilepsy patients [31]. In our study, an increased expression of APOA4 was found in the epilepsy group. The overall overexpression of proteins involved in lipid metabolism was established in our study, which is in agreement with previous studies of TLE.

During the study, overrepresented proteins with antioxidant activity were identified, which may be a compensatory reaction for oxidative stress. Among them are CP, AFM, PON1, PRDX2, RBP4, BCHE, HPX, SELENOP, and CFHR4. The attenuation of oxidative damage in models of acquired epilepsy appears to protect against cognitive malfunction, as well as provide neuroprotection [32].

### 3.4. Neuroinflammation

The impaired activation and regulation of inflammatory cells and molecules in damaged nervous tissue is one of the factors in the development and maintenance of epileptogenic activity. Status epilepticus can activate micro- and astroglia, as well as cells of the peripheral immune system, to release several pro-inflammatory mediators, thereby initiating a cascade of inflammatory processes in brain tissue, which, in turn, can change the excitability of neurons and affect the physiological functions of glia.

Patients with both MRI-positive and MRI-negative forms of epilepsy had clinical and electroencephalographic manifestations of TLE, according to the International League Against Epilepsy (ILAE) criteria. Inflammation and other immune-mediated processes are essential in epileptogenesis [33]. Clinically, brain injury activates innate immunity, and the disruption of the blood–brain barrier allows adaptive immunity and peripheral immune responses, as well as specific antibody production, to influence the brain. These mechanisms contribute to epileptogenesis in many acquired epilepsies. Processes of neuroinflammation are equally identified in many acquired models of epilepsy (e.g., after status epilepticus, stroke, and traumatic brain injuries) [34,35].

Several proteins that induce inflammatory processes were found to be upregulated in epilepsy in our study. These are C9, C1R, FGG, ITIH1, ITIH2, ITIH4, ATRN, SPP24, F5, C8A, SELL, AGT, CFD, CNDP1, FCN3, and FCN2.

The neuroinflammatory process is accompanied by oxidative stress. Oxidative stress is mediated by the formation of reactive oxygen species (ROS), which plays an important role in the pathogenesis of epilepsy [36]. Recurrent epileptic seizures can increase the concentration of reactive oxygen species and superoxides in the brain [37]. Free radicals can directly induce seizure activity through the inactivation of glutamine synthase, which leads to glutamate excitotoxicity [38], as well as the inhibition of glutamate decarboxylase. In patients with TLE, antioxidant systems such as glutathione and superoxide dismutase (SOD) are altered, indicating ongoing oxidative stress [39].

The complement system is involved in the epileptogenesis of TLE in the chronic phase, both in experimental models and in humans [40]. Persistent complement activation can promote a sustained inflammatory response and destabilize the neural networks involved. The sequential administration of five membrane attack pathway proteins into the hippocampus of awake and free-moving rats induced both behavioral and electrographic seizures, as well as cytotoxicity. The onset of seizures occurred during or shortly after the C8/C9 infusion [41]. The overexpression of C8B was found in the plasma of patients with Rolandic epilepsy [10]. The overexpression of C9 was observed in the hippocampal tissues of patients with TLE [40]. Our study found increased C9 expression in the epilepsy group. Elevated levels of C8 and C8A were also found in the group of MRI-positive patients in comparison with the MRI-negative ones, which indicates a more intensive neuroinflammatory process in MRI-positive patients.

Angiotensin (AGT) promotes an inflammatory response causing oxidative stress. AGT receptor activation has been demonstrated in the cortex and hippocampus of mesial temporal sclerosis [42]. AGT mediates a predisposition to seizures [43]. It has an inhibitory effect on K^+^-induced GABA release, as demonstrated by the analysis of hippocampal sections [44]. Our study demonstrated AGT overexpression in the epilepsy group compared to the healthy group.

The inflammatory process is compensated by the overexpression of proteins, in one way or another, inhibiting inflammatory cascades. Our study found several proteins that compensate for the inflammatory response, including SPP24, A2M, CLU, CPN2, CPB2, FETUB, VASN, C4BPB, IGLC1, IGKV3, IGKV4-1, IGKC, PROZ, PROC, VTN, IGFALS, BTD, AFM, and AAG.

Alpha-2-macroglobulin (A2M) is a glycoprotein that can inhibit proteases without directly blocking their active site. It suppresses complement activation. An increased content of A2M in the CSF was found in children with encephalitis [45]. Moderately elevated levels of A2M were also observed in the CSF of patients with drug-resistant epilepsy [46]. In our study, the increased expression of A2M was observed in the MRI-negative group compared to the MRI-positive group.

Clusterin (CLU or APOJ) exhibits a protective effect against brain damage by stabilizing stress proteins and inhibiting apoptosis. Traumatic brain injuries (TBIs) induce massive CLU mRNA expression and immunoreactivity in neurons and astroglia [47]. Clusterin knockout leads to increased proapoptotic activity in neurons [48]. Induced status epilepticus leads to a sharp increase in clusterin mRNA in glial cells. The increased expression of this protein may be a compensatory response aimed at slowing down the neurodegenerative cascade controlled by the complement system [49]. An increased expression of CLU was observed in the TLE patients, where it may be a response to medication by valproic acid [50].

Vitronectin (VTN) is a multifunctional glycoprotein of the hemopexin family, present in the blood and extracellular matrix. It binds glycosaminoglycans, collagen, and plasminogen, which are involved in neurodegenerative processes. It is an indicator of the reactive gliosis of the hippocampus, which was demonstrated in kainic models of epileptogenesis [51]. An increased VTN expression was observed in MRI-positive patients compared to MRI-negative patients, which was noted in previous studies.

Gelsolin (GSN) is associated with the reorganization of actin in microglia, which contributes to the restoration of the processes of the nervous tissue after damage and inflammation. Studies of *GSN* knockout mice [52] showed that gelsolin protects neurons from excitotoxic Ca^2+^ overload in vitro and in vivo. A marked decrease in GSN levels is observed in CSF and tissue in patients with TLE [53]. In our study, on the contrary, an increased expression of GSN was observed in the whole group of epilepsy in comparison with the healthy group, which may be caused by compensatory mechanisms.

### 3.5. Signal Transduction

Neuroinflammation and lipid peroxidation can disrupt the membrane structure, leading to a change in cell permeability, by increasing the viscosity of the membrane, which, in turn, can trigger a cascade of events leading to a change in the functioning of membrane proteins and the dysregulation of intracellular signaling proteins, which further results in the increased excitability of neurons. Our study revealed an upregulation in epilepsy of a significant number of serpin proteins involved in the control of intracellular signaling. These are SERPINA3, SERPINF1, SERPINA10, SERPING1, SERPIND1, SERPINC1, and SERPINF2, which confirms the hypothesis of the massive dysregulation of intracellular processes. The neuroinflammatory process and the accompanying cascade of molecular events ultimately lead to disturbances in the structure of cell membranes and membrane proteins, which contribute to the spread and maintenance of epileptogenic activity. In the aberrant expression of proteins involved in various biological, cellular, and molecular processes, the systemic nature of the disease is observed. The presence of similar aberrations in several other neurological disorders indicates the association of TLE with other brain diseases.

### 3.6. Antiepileptic Drugs’ Influence

The majority of studied patients (88.9%) received antiepileptic drugs (AEDs) recommended for the treatment of focal seizures and focal bilateral tonic clonic seizures (FBTCs). Antiepileptic therapy should obviously affect the protein expression. Thus, an increased expression of some specific proteins was found in the epilepsy group. These are HPX, CP, BCHE, PON1, CLU (APOJ), FGG, APOA1, BTD, and immunoglobulins, as well as proteins of the coagulation cascade. According to the previous research, some of the protein-level alterations that we have been discovered may be a consequence of taking antiepileptic drugs. In particular, HPX expression can be induced by phenobarbital [54]; the expression of CP, PON1, APOA1, and FGG can be influenced by valproic acid [55,56,57]. Therefore, these proteins cannot be considered to be potential biomarkers. Additional data on the possible effect of drugs on protein expression are presented in Table S4.

### 3.7. Limitations and Future Directions

This study was focused on the primary comparison of proteomes of blood plasma from epilepsy patients, including patients with structural TLE and focal TLE of unspecified etiology (MRI-negative), and healthy individuals. This selection of study groups initially has a number of limitations. The main restriction is the limited sample set available at one time. The complications were related to the sample collection from the patients meeting the inclusion criteria, laborious sample preparation, and reproducibility limitations during the analysis and processing of the sample set. Moreover, most of the involved patients were taking AEDs, which certainly can influence the protein expression. We were unable to overcome the difficulty in identifying proteins that are strictly unaffected by drugs due to the low availability of untreated patients.

Our study had the general purpose of a primary search. The focus on studying individual proteins suggested as biomarker candidates and revealing their functions in the mechanism of epilepsy development requires special targeted research, which was beyond our purpose. Future work should include targeted proteomic studies of large patient cohorts for the validation of the presented results in terms of reliability and generalizability.

## 4. Materials and Methods

### 4.1. Patient Cohort

All participants signed an informed consent. The study was approved by the Local Ethics Committee of Prof. V.F. Voino-Yasenetsky Krasnoyarsk State Medical University Ministry of Health of Russia (protocol No. 85/2018 dated 27 September 2018). Blood samples were collected in the Neurological Center for Epileptology, Neurogenetics, and Brain Research of the University Clinics, Prof. V.F. Voino-Yasenetsky Krasnoyarsk State Medical University, Krasnoyarsk, Russia.

The entire study cohort included 34 individuals. Plasma samples were collected from 27 patients with temporal lobe epilepsy of various origins and 7 healthy volunteers. The epilepsy cohort was divided into MRI-negative and MRI-positive groups, including three types of lesions: hippocampus sclerosis, vascular anomaly, and malformations (Table 1).

Patients met the following criteria for inclusion in the study: (1) absence of acute infectious diseases at the time of the study and within 1 month before blood sampling, (2) absence of somatic and mental illnesses in the stage of decompensation, (3) absence of any comorbidities potentially affecting the plasma proteome, (4) no hereditary history of epilepsy, (5) taking antiepileptic drugs (AEDs) not exceeding the average therapeutic daily dose, (6) absence of clinical signs of side effects of AEDs at the time of the study, and (7) absence of focal bilateral tonic clonic seizures (FBTCs) within 4 weeks before the study.

We found 25 out of 27 (88.9%) patients took AEDs, and 12 out of 25 (48%) patients received AED monotherapy. The patients predominantly received sodium channel blockers, both in monotherapy and in combination with other AEDs. The patients were divided into drug-resistant and drug-responsive groups according to the International League Against Epilepsy (ILAE) criteria (failure of adequate trials of two tolerated and appropriately chosen AED schedules (whether as monotherapies or in combination) to achieve seizure freedom).

The median duration of epilepsy was 13 (7–26) years. In 6 out of 27 (22.2%) cases, remission of epileptic seizures was observed, lasting from 1 to 6 years, with an average of 2.8 years, against the background of ongoing therapy with anticonvulsants. Remission and without-study-limitation FBTCs were recorded during the last year in 8 out of 27 (29.6%) patients, with an average frequency of 4.9 attacks per year. FBTCs were absent from patients within 1 month before inclusion in the study. In 18 patients, seizures with a focal onset, motor and non-motor, with and without impaired awareness, were recorded, with an average frequency of 2.6 months.

### 4.2. Blood Sample Treatment

The blood samples were collected into heparin vacuum tubes (BD, Franklin Lakes, NJ, USA) and centrifuged for 10 min at 3000 rpm; collected plasma was stored at −80 °C. The thawed plasma samples were depleted from albumin and immunoglobulin using ProteoPrep Blue Albumin & IgG Depletion Kit (Millipore, Darmstadt, Germany) according to the manufacturer protocol. Sample preparation for mass spectrometry was carried out as described earlier [58]. Briefly, the depleted plasma samples containing 4 micrograms of protein were reduced by dithiothreitol, alkylated by iodoacetamide, and digested by trypsin according to the manufacturer protocol (Thermo Scientific, Waltham, MA, USA). Then, the samples were desalted by C18 pipette tips from the same manufacturer under its protocol and dried. All plasma samples were prepared in triplicates.

### 4.3. Mass Spectrometry

The samples were dissolved in water containing 0.1% of formic acid (Sigma-Aldrich, St. Louis, MO, USA), and injected into the Dionex UltiMate 3000 RSLC nano liquid chromatographer (Thermo Scientific, Waltham, MA, USA). Peptides were separated on an in-house packed reversed-phase C18 column (Polymicro Technology, Phoenix, AZ, USA), 15 cm × 70 μm ID, Luna C18, 3 μm, 100 Å (Phenomenex, Torrance, CA, USA), using a standard 0–38% water/acetonitrile/0.1% formic acid gradient at the flow rate of 300 nL/min. The Orbitrap Fusion mass spectrometer (Thermo Scientific, Waltham, MA, USA) was operated in data-dependent mode with scans of precursor and fragment ions changing in a cycle of 3 s. Precursor ions were detected at a resolution of 60,000 by the Orbitrap. The fragment ions were generated by collision-induced dissociation (CID) at 35% of collision energy and were registered by an ion trap detector.

### 4.4. Data Analysis

The full set of RAW data files obtained by mass spectrometry was processed using MaxQuant version 2.0 proteomic software. The label-free quantification (LFQ) parameter was enabled. A protein search was performed using the actual annotated UniProt database available online: https://www.uniprot.org/help/downloads/ (accessed on 18 July 2024) with the false discovery rate (FDR) set to 0.01. The obtained LFQ values for each identified protein were considered as relative quantitative measures of protein distribution among the plasma samples.

The tripled LFQ data for each plasma sample were averaged using weights corresponding to the fraction of proteins that lead in a certain triplicate. Principal component analysis (PCA) was applied to the obtained LFQ dataset to assess primary proteomic differences between the groups using the SIMCA version 13 program (Sartorius AG, Goettingen, Germany). A general comparison of protein distributions among the selected groups to identify specific biomarker candidates was carried out using Welch’s *t*-test with the Benjamini–Hochberg correction for multiple comparisons. FDR was set to 0.01. Analysis was performed using Anaconda Distribution Python version 3.8. The analysis of biological functions, as well as the drawing of the corresponding diagrams, was carried out using the SninyGo version 0.76 program available online: http://bioinformatics.sdstate.edu/go/ (accessed on 18 July 2024).

## 5. Conclusions

Epilepsy can be caused by various reasons, but, finally, they all lead to the onset and persistence of the inflammatory process caused and maintained by several proteins found in our study, which, in turn, triggers a molecular cascade of events that contribute to the spread and preservation of epileptogenic activity. Neuroinflammation and the accompanying oxidative stress can be compensated to some extent by the induction of inflammation-inhibiting proteins and antioxidants. However, uncompensated processes ultimately lead to the dysregulation of the functions of the membrane and membrane proteins, which results in a decrease in the activation threshold of channels and the spread of excitation.

Plasma proteome profiling may be a way to identify potential biomarkers of epileptogenesis and/or seizure activity. While some of the most differentially regulated proteins found in our study are new, such as protein transport agent afamin (AFM); phosphatidylcholine-sterol acyltransferase (LCAT), which is responsible for the extracellular metabolism of plasma lipoproteins; and some protease inhibitors from the serpin family, most of the others have previously been reported in the studies of epilepsy. These proteins may ultimately help to explore pathways of epileptogenesis, while the further validation of new potential candidates in large patient cohorts versus other neurological disorders will confirm their use as potential biomarkers for clinical diagnosis.

## Figures and Tables

**Table 1:** Epilepsy cohort.

N	ID	G	Age over 40	GD	MRI Status	DR	FBTC	Freq. of FBTC, per Month	Last FBTC, Year	Duration of Epilepsy, Years	Antiepileptic Drug, mg/per Day	Focal Seizure Freq., per Month	Duration of Remission, Years
1	A1	F	No	TLE	Neg.	R	Yes	7	2021	29	LTG 200 mg + TPM 100 mg	7	No
2	A2	M	Yes	TLE	Neg.	R	Yes	3	2021	11	LCS 150 mg + LEV 1500 mg	No	No
3	A3	M	No	TLE	Neg.	R	Yes	12	2021	10	LTG 150 mg	No	No
4	V1	F	Yes	TLE	Neg.	NR	Yes	2	2021	1	OXC 600 mg	No	No
5	V2	F	No	TLE	Neg.	R	RE		2015	24	No	No	6
6	V3	F	No	TLE	Neg.	NR	RE		2017	13	LCS 100 mg	No	3
7	V4	F	No	TLE	Neg.	R	RE		2018	3	LCS 75 mg	No	3
8	V5	M	No	TLE	Neg.	NR	RE		2015	25	CBZ 400 mg	No	2
9	V6	F	No	TLE	Neg.	NR	No		2019	27	CBZ 800 mg + LEV 1000 mg	No	2
10	VA	F	No	TLE & M	Neg.	R	No		2020	18	CBZ 800 mg	No	1
11	V7	F	No	TLE & S	Neg.	R	Yes	1	2020	6	LTG 150 mg + VPA 450 mg + PMP 4 mg	<1	No
12	VG1	F	Yes	TLE & S	Pos.	R	RE	12	2020	48	LTG 200 mg + PMP 4 mg	6	No
13	VG2	F	No	TLE & S	Pos.	R	No			12	LCS 350 mg	5	No
14	VG3	M	No	TLE & S	Pos.	NRt	Yes	1	2020	4	LCS 200 mg	1	No
15	VG4	F	No	TLE & S	Pos.	R	No			9	CBZ 600 mg	1	No
16	VG5	F	No	TLE & S	Pos.	NR	Yes		2019	32	CBZ 300 mg	2	No
17	VG6	F	No	TLE & S	Pos.	NR	RE		2017	22	VPA 200 mg + LTG 200 mg + ZNS 300 mg	4	No
18	VG7	M	Yes	TLE & S	Pos.	R	RE		2018	46	PB 175 mg + LCS 200 mg + VPA 600 mg	2	No
19	VG8	M	Yes	TLE & S	Pos.	R	No			5	LEV 2000 mg + OXC 2100 mg	1	No
20	VS1	M	No	TLE & VA	Pos.	NR	Yes	1	2020	11	CBZ 1000 mg + LTG 200 mg	2	No
21	VS2	F	Yes	TLE & VA	Pos.	R	RE		2013	28	VPA 900 mg + OXC 600 mg	6	No
22	VS3	M	Yes	TLE & VA	Pos.	R	RE		2017	46	LTG 150 mg + VPA 900 mg + LEV 1500 mg	2	No
23	VP1	F	Yes	TLE & M	Pos.	NR	No			15	OXC 600 mg	2	No
24	VP2	M	No	TLE & M	Pos.	R	No			8	OXC 600 mg	1	No
25	VP3	F	Yes	TLE & M	Pos.	NR	No		2020	3	No	1	No
26	VPG1	M	Yes	TLE & M & S	Pos.	R	RE		2018	4	OXC 600 mg + BRV 100 mg	2	No
27	VPG2	M	No	TLE & M & S	Pos.	R	RE		2010	17	OXC 1500 mg + LEV 1500 mg + LCS 400 mg	1	No

G—gender (M—male; F—female). GD—general diagnosis (TLE—temporal lobe epilepsy; TLE & M—temporal lobe epilepsy with malformations; TLE & S—temporal lobe epilepsy with hippocampus sclerosis; TLE & VA—temporal lobe epilepsy vascular anomaly; TLE & M & S—temporal lobe epilepsy with malformations and hippocampus sclerosis). DR—drug-resistance (R—resistant; NR—non-resistant). FBTC—focal bilateral tonic clonic seizure (RE—registered earlier). Antiepileptic Drugs: BRV—Brivaracetam; CBZ—Carbamazepine; LCS—Lacosamide; LEV—Levetiracetam; LTG—Lamotrigine; OXC—Oxcarbazepine; PB—Phenobarbital; PMP—Perampanel; TPM—Topiramate; VPA—Valproate; ZNS—Zonisamide.

**Table 2:** Upregulated epilepsy proteins involved in different biological processes.

Number of Involved Proteins	High-Level Gene Ontology Category	Protein’s Names
26	Response to stress	CPB2, ATRN, SERPIND1, HPX, APOA4, C9, SERPINC1, APOA1, CLU, C4BPB, PROZ, SERPINF1, AGT, GSN, SERPING1, ALB, PRDX2, FGG, A2M, APOD, SERPINA3, SERPINA1, ORM1, HPR, SERPINF2, SERPINA10
20	Response to external stimulus	PON1, CPB2, SERPIND1, HPX, APOA4, C9, BCHE, SERPINC1, APOA1, CLU, C4BPB, SERPINF1, AGT, GSN, SERPING1, ALB, PRDX2, FGG, A2M, SERPINF2
20	Regulation of molecular function	PON1, ITIH1, SPP2, SERPIND1, APOA4, SERPINC1, APOA1, CLU, SERPINF1, AGT, GSN, SERPING1, PRDX2, A2M, CPN2, SERPINA3, SERPINA1, APOC2, SERPINF2, SERPINA10
19	Immune system process	CPB2, HPX, APOA4, C9, SERPINC1, APOA1, CLU, C4BPB, GSN, SERPING1, PRDX2, FGG, A2M, CPN2, SELL, APOD, SERPINA3, SERPINA1, ORM1
18	Regulation of response to stimulus	CPB2, HPX, C9, SERPINC1, APOA1, CLU, C4BPB, SERPINF1, AGT, SERPING1, PRDX2, FGG, A2M, CPN2, SELL, APOD, VASN, SERPINF2
17	Immune response	CPB2, HPX, APOA4, C9, SERPINC1, APOA1, CLU, C4BPB, GSN, SERPING1, FGG, A2M, CPN2, SELL, SERPINA3, SERPINA1, ORM1
14	Regulation of the immune system process	CPB2, HPX, C9, APOA1, CLU, C4BPB, SERPING1, PRDX2, FGG, A2M, CPN2, SELL, APOD, ORM1
11	Regulation of signaling	HPX, BCHE, APOA1, CLU, AGT, PRDX2, FGG, A2M, APOD, VASN, SERPINF2
8	Catabolic process	PON1, APOA4, APOA1, CLU, C4BPB, ALB, PRDX2, APOC2
7	Activation of immune response	CPB2, C9, CLU, C4BPB, SERPING1, A2M, CPN2
7	Regulation of hemostasis	CPB2, SERPINC1, SERPING1, PRDX2, FGG, A2M, SERPINF2
6	Regulation of plasma lipoprotein particle levels	APOA4, APOA1, AGT, ALB, LCAT, APOC2

## Data Availability

The original data are available upon reasonable request from the corresponding authors.

## Supplementary Materials

The following supporting information can be downloaded at: https://www.mdpi.com/article/10.3390/ijms25147935/s1.

## References

[1] Thijs R.D., Surges R., O’Brien T.J., Sander J.W. (2019). Epilepsy in adults. Lancet.

[2] Blümcke I., Thom M., Aronica E., Armstrong D.D., Bartolomei F., Bernasconi A., Bernasconi N., Bien C.G., Cendes F., Coras R. (2013). International consensus classification of hippocampal sclerosis in temporal lobe epilepsy: A Task Force report from the ILAE Commission on Diagnostic Methods. Epilepsia.

[3] Engel J., Pitkänen A. (2020). Biomarkers for epileptogenesis and its treatment. Neuropharmacology.

[4] Moshé S.L., Perucca E., Ryvlin P., Tomson T. (2015). Epilepsy: New advances. Lancet.

[5] Abraira L., Santamarina E., Cazorla S., Bustamante A., Quintana M., Toledo M., Fonseca E., Grau-López L., Jiménez M., Ciurans J. (2020). Blood biomarkers predictive of epilepsy after an acute stroke event. Epilepsia.

[6] Monti G., Tondelli M., Giovannini G., Bedin R., Nichelli P.F., Trenti T., Meletti S., Chiari A. (2015). Cerebrospinal fluid tau proteins in status epilepticus. Epilepsy Behav..

[7] Zhu H., Snyder M. (2002). “Omic” approaches for unraveling signaling networks. Curr. Opin. Cell Biol..

[8] Ashton N.J., Nevado-Holgado A.J., Barber I.S., Lynham S., Gupta V., Chatterjee P., Goozee K., Hone E., Pedrini S., Blennow K. (2019). A plasma protein classifier for predicting amyloid burden for preclinical Alzheimer’s disease. Sci. Adv..

[9] Bitsika V., Duveau V., Simon-Areces J., Mullen W., Roucard C., Makridakis M., Mermelekas G., Savvopoulos P., Depaulis A., Vlahou A. (2016). High-Throughput LC-MS/MS Proteomic Analysis of a Mouse Model of Mesiotemporal Lobe Epilepsy Predicts Microglial Activation Underlying Disease Development. J. Proteome Res..

[10] Sun J., Jiang T., Gu F., Ma D., Liang J. (2020). TMT-Based Proteomic Analysis of Plasma from Children with Rolandic Epilepsy. Dis. Markers.

[11] Panina Y.S., Timechko E.E., Usoltseva A.A., Yakovleva K.D., Kantimirova E.A., Dmitrenko D.V. (2023). Biomarkers of Drug Resistance in Temporal Lobe Epilepsy in Adults. Metabolites.

[12] Alvim M.K.M., Morita-Sherman M.E., Yasuda C.L., Rocha N.P., Vieira É.L., Pimentel-Silva L.R., Henrique Nogueira M., Barbosa R., Watanabe N., Coan A.C. (2021). Inflammatory and neurotrophic factor plasma levels are related to epilepsy independently of etiology. Epilepsia.

[13] Kim I., Mlsna L.M., Yoon S., Le B., Yu S., Xu D., Koh S. (2015). A postnatal peak in microglial development in the mouse hippocampus is correlated with heightened sensitivity to seizure triggers. Brain Behav..

[14] Sinha S., Patil S.A., Jayalekshmy V., Satishchandra P. (2008). Do cytokines have any role in epilepsy?. Epilepsy Res..

[15] Gorter J.A., Van Vliet E.A., Aronica E. (2015). Status epilepticus, blood–brain barrier disruption, inflammation, and epileptogenesis. Epilepsy Behav..

[16] Oby E., Janigro D. (2006). The Blood–Brain Barrier and Epilepsy. Epilepsia.

[17] Webster K.M., Sun M., Crack P., O’Brien T.J., Shultz S.R., Semple B.D. (2017). Inflammation in epileptogenesis after traumatic brain injury. J. Neuroinflammation.

[18] Dudek F.E., Staley K.J., Noebels J.L., Avoli M., Rogawski M.A., Olsen R.W., Delgado-Escueta A.V. (2012). The Time Course and Circuit Mechanisms of Acquired Epileptogenesis. Jasper’s Basic Mechanisms of the Epilepsies.

[19] Williams P.A., White A.M., Clark S., Ferraro D.J., Swiercz W., Staley K.J., Dudek F.E. (2009). Development of spontaneous recurrent seizures after kainate-induced status epilepticus. J. Neurosci..

[20] Kadam S.D., White A.M., Staley K.J., Dudek F.E. (2010). Continuous electroencephalographic monitoring with radio-telemetry in a rat model of perinatal hypoxia-ischemia reveals progressive post-stroke epilepsy. J. Neurosci..

[21] Pitkänen A., Lukasiuk K. (2011). Mechanisms of epileptogenesis and potential treatment targets. Lancet Neurol..

[22] Blume W.T. (2006). The Progression of Epilepsy. Epilepsia.

[23] Yang J.-W., Czech T., Gelpi E., Lubec G. (2005). Extravasation of plasma proteins can confound interpretation of proteomic studies of brain: A lesson from apo A-I in mesial temporal lobe epilepsy. Brain Res. Mol. Brain Res..

[24] Farooqui A.A., Yang H.C., Horrocks L. (1997). Involvement of phospholipase A2 in neurodegeneration. Neurochem. Int..

[25] Ong W.Y., He Y., Suresh S., Patel S.C. (1997). Differential expression of apolipoprotein D and apolipoprotein E in the kainic acid-lesioned rat hippocampus. Neuroscience.

[26] Montpied P., de Bock F., Lerner-Natoli M., Bockaert J., Rondouin G. (1999). Hippocampal alterations of apolipoprotein E and D mRNA levels in vivo and in vitro following kainate excitotoxicity. Epilepsy Res..

[27] Rassart E., Bedirian A., Do Carmo S., Guinard O., Sirois J., Terrisse L., Milne R. (2000). Apolipoprotein D. Biochim. Biophys. Acta.

[28] Andersen J.K. (2004). Oxidative stress in neurodegeneration: Cause or consequence?. Nat. Med..

[29] Reindl M., Knipping G., Wicher I., Dilitz E., Egg R., Deisenhammer F., Berger T. (2001). Increased intrathecal production of apolipoprotein D in multiple sclerosis. J. Neuroimmunol..

[30] Kumar A., Tripathi M., Pandey R.M., Ramakrishnan L., Srinivas M., Luthra K. (2006). Apolipoprotein E in temporal lobe epilepsy: A case-control study. Dis. Markers.

[31] Saengow V.E., Chiangjong W., Khongkhatithum C., Changtong C., Chokchaichamnankit D., Weeraphan C., Kaewboonruang P., Thampratankul L., Manuyakorn W., Hongeng S. (2021). Proteomic analysis reveals plasma haptoglobin, interferon-γ, and interleukin-1β as potential biomarkers of pediatric refractory epilepsy. Brain Dev..

[32] Pearson J.N., Rowley S., Liang L.-P., White A.M., Day B.J., Patel M. (2015). Reactive oxygen species mediate cognitive deficits in experimental temporal lobe epilepsy. Neurobiol. Dis..

[33] Vezzani A., French J., Bartfai T., Baram T.Z. (2011). The role of inflammation in epilepsy. Nat. Rev. Neurol..

[34] Lehrmann E., Guidetti P., Löve A., Williamson J., Bertram E.H., Schwarcz R. (2008). Glial activation precedes seizures and hippocampal neurodegeneration in measles virus–infected mice. Epilepsia.

[35] Stewart K.-A.A., Wilcox K.S., Fujinami R.S., White H.S. (2010). Development of Postinfection Epilepsy After Theiler’s Virus Infection of C57BL/6 Mice. J. Neuropathol. Exp. Neurol..

[36] Calik M., Oguz E., Sarikaya S., Kocaturk O., Koca B., Gungor H.E., Aksoy N., Yoldas T.K., Iscan A. (2014). An evaluation of serum paraoxonase together with arylesterase activities and oxidative stress in children with intractable epilepsy: A cross-sectional study. Epilepsy Res..

[37] Oliver C.N., Starke-Reed P.E., Stadtman E.R., Liu G.J., Carney J.M., Floyd R.A. (1990). Oxidative damage to brain proteins, loss of glutamine synthetase activity, and production of free radicals during ischemia/reperfusion-induced injury to gerbil brain. Proc. Natl. Acad. Sci. USA.

[38] Reynolds I.J., Hastings T.G. (1995). Glutamate induces the production of reactive oxygen species in cultured forebrain neurons following NMDA receptor activation. J. Neurosci..

[39] Ristić A.J., Savić D., Sokić D., Bogdanović Pristov J., Nestorov J., Baščarević V., Raičević S., Savić S., Spasojević I. (2015). Hippocampal antioxidative system in mesial temporal lobe epilepsy. Epilepsia.

[40] Aronica E., Boer K., van Vliet E.A., Redeker S., Baayen J.C., Spliet W.G.M., van Rijen P.C., Troost D., da Silva F.H.L., Wadman W.J. (2007). Complement activation in experimental and human temporal lobe epilepsy. Neurobiol. Dis..

[41] Xiong Z.-Q., Qian W., Suzuki K., McNamara J.O. (2003). Formation of complement membrane attack complex in mammalian cerebral cortex evokes seizures and neurodegeneration. J. Neurosci..

[42] Argañaraz G.A., Konno A.C., Perosa S.R., Santiago J.F.C., Boim M.A., Vidotti D.B., Varella P.P.V., Costa L.G., Canzian M., Porcionatto M.A. (2008). The renin-angiotensin system is upregulated in the cortex and hippocampus of patients with temporal lobe epilepsy related to mesial temporal sclerosis. Epilepsia.

[43] Georgiev V., Petkova B., Kambourova T. (1988). Adaptive changes in the effects of angiotensin II on the convulsive-seizure threshold in cases of altered sensitivity of dopamine receptors. Methods Find. Exp. Clin. Pharmacol..

[44] Hadjiivanova C.H., Georgiev V. (1998). In vitro effect of angiotensin II on GABA release in rat hippocampus. Neuropeptides.

[45] Suzuki Y., Hashimoto K., Hoshi K., Ito H., Kariya Y., Miyazaki K., Sato M., Kawasaki Y., Yoshida M., Honda T. (2019). Ratio of Alpha 2-Macroglobulin Levels in Cerebrospinal Fluid and Serum: An Expression of Neuroinflammation in Acute Disseminated Encephalomyelitis. Pediatr. Neurol..

[46] Vasil’eva T.G., Dobrogorskaia L.N., Kraeva L.N., Goncharova V.P. (1989). Alpha-2 macroglobulin from cerebrospinal fluid in neurosurgical diseases. Vopr. Med. Khim.

[47] Imhof A., Charnay Y., Vallet P.G., Aronow B., Kovari E., French L.E., Bouras C., Giannakopoulos P. (2006). Sustained astrocytic clusterin expression improves remodeling after brain ischemia. Neurobiol. Dis..

[48] Wehrli P., Charnay Y., Vallet P., Zhu G., Harmony J., Aronow B., Tschopp J., Bouras C., Viard-Leveugle I., French L.E. (2001). Inhibition of post-ischemic brain injury by clusterin overexpression. Nat. Med..

[49] May P.C., Lampert-Etchells M., Johnson S.A., Poirier J., Masters J.N., Finch C.E. (1990). Dynamics of gene expression for a hippocampal glycoprotein elevated in Alzheimer’s disease and in response to experimental lesions in rat. Neuron.

[50] Nuutinen T., Suuronen T., Kauppinen A., Salminen A. (2010). Valproic acid stimulates clusterin expression in human astrocytes: Implications for Alzheimer’s disease. Neurosci. Lett..

[51] Niquet J., Gillian A., Ben-Ari Y., Represa A. (1996). Reactive glial cells express a vitronectin-like protein in the hippocampus of epileptic rats. Glia.

[52] Furukawa K., Fu W., Li Y., Witke W., Kwiatkowski D.J., Mattson M.P. (1997). The actin-severing protein gelsolin modulates calcium channel and NMDA receptor activities and vulnerability to excitotoxicity in hippocampal neurons. J. Neurosci..

[53] Peng X., Zhang X., Wang L., Zhu Q., Luo J., Wang W., Wang X. (2011). Gelsolin in cerebrospinal fluid as a potential biomarker of epilepsy. Neurochem. Res..

[54] Tutor J.C., Fernandez M.P., Paz J.M. (1982). Serum copper concentration and hepatic enzyme induction during long-term therapy with anticonvulsants. Clin. Chem..

[55] Lampón N., Tutor J.C. (2010). Effect of valproic acid treatment on copper availability in adult epileptic patients. Clin. Biochem..

[56] Eirís J., Novo-Rodríguez M., Del Río M., Meseguer P., Del Río M.C., Castro-Gago M. (2000). The effects on lipid and apolipoprotein serum levels of long-term carbamazepine, valproic acid and phenobarbital therapy in children with epilepsy. Epilepsy Res..

[57] Larsson P., Alwis I., Niego B., Sashindranath M., Fogelstrand P., Wu M.C.L., Glise L., Magnusson M., Daglas M., Bergh N. (2016). Valproic acid selectively increases vascular endothelial tissue-type plasminogen activator production and reduces thrombus formation in the mouse. J. Thromb. Haemost..

[58] Glazyrin Y.E., Veprintsev D.V., Ler I.A., Rossovskaya M.L., Varygina S.A., Glizer S.L., Zamay T.N., Petrova M.M., Minic Z., Berezovski M.V. (2020). Proteomics-Based Machine Learning Approach as an Alternative to Conventional Biomarkers for Differential Diagnosis of Chronic Kidney Diseases. Int. J. Mol. Sci..

