# Supplementary material for: Comparative Proteomic Profiling of Blood Plasma Revealed Marker Proteins Involved in Temporal Lobe Epilepsy
Source: Int J Mol Sci. 2024 Jul 20;25(14):7935. doi: 10.3390/ijms25147935 (PMC11276668; doi:10.3390/ijms25147935)
Supplement: Supplementary file 1 [file ijms-25-07935-s001.zip › Table S1 REVISED.pdf]

Table S1. Significantly upregulated proteins for epilepsy group.

Gene ID	Protein ID	Protein name	P-value	Corrected p-value
HPX	P02790	Hemopexin	4.35E-12	8.36143E-10
LCAT	P04180	Phosphatidylcholine-sterol acyltransferase	1.44E-08	1.37837E-06
CP	P00450	Ceruloplasmin	5.36E-08	3.42853E-06
SERPINA3	P01011	Alpha-1-antichymotrypsin	9.2E-08	4.4177E-06
SERPINF1	P36955	Pigment epithelium-derived factor	2.07E-07	7.95021E-06
AFM	P43652	Afamin	2.59E-07	8.28641E-06
APOA1	P02647	Apolipoprotein A-I	5.03E-07	1.37921E-05
A2M	P01023	Alpha-2-macroglobulin	8.47E-07	2.03292E-05
APOD	P05090	Apolipoprotein D	1.74E-06	3.71366E-05
SERPING1	P05155	Plasma protease C1 inhibitor	6.58E-06	0.00012626
HPR	P00739	Haptoglobin-related protein	1.32E-05	0.000229792
APOA4	P06727	Apolipoprotein A-IV	1.63E-05	0.000260171
SPP2	Q13103	Secreted phosphoprotein 24	3.36E-05	0.000496895
SERPINA1	P01009	Alpha-1-antitrypsin	4.33E-05	0.00059392
FGG	P02679	Fibrinogen gamma chain	6.43E-05	0.000822973
APOC2	P02655	Apolipoprotein C-II	0.000107	0.00128207
IGKC	P01834	Immunoglobulin kappa constant	0.000164	0.001849287
BCHE	P06276	Cholinesterase	0.000173	0.001849287
SERPIND1	P05546	Heparin cofactor 2	0.000187	0.001893896
CLU	P10909	Clusterin	0.000261	0.002503569
CPN2	P22792	Carboxypeptidase N subunit 2	0.000281	0.002564806
ITIH1	P19827	Inter-alpha-trypsin inhibitor heavy chain H1	0.000294	0.002564806
CPB2	Q96IY4	Carboxypeptidase B2	0.000353	0.002943201
C9	P02748	Complement component C9	0.000417	0.003136348
SELL	P14151	L-selectin	0.000425	0.003136348
C4BPB	P20851	C4b-binding protein beta chain	0.000502	0.003445611
PON1	P27169	Serum paraoxonase/arylesterase 1	0.000502	0.003445611
SERPINC1	P01008	Antithrombin-III	0.000539	0.00345882
C1R	P00736	Complement C1r subcomponent	0.00054	0.00345882
BTD	P43251	Biotinidase	0.000571	0.003534305
AGT	P01019	Angiotensinogen	0.000594	0.003566911
VASN	Q6EMK4	Vasorin	0.000713	0.004147724
PROZ	P22891	Vitamin K-dependent protein Z	0.001082	0.005937493
IGKV4-1	P06312	Immunoglobulin kappa variable 4-1	0.001131	0.006031396
ORM1	P02763	Alpha-1-acid glycoprotein 1	0.001227	0.006364929
ATRN	O75882	Attractin	0.001275	0.00644353
IGL1	P0DOX8	Immunoglobulin lambda-1 light chain	0.001322	0.006507876
SERPINA10	Q9UK55	Protein Z-dependent protease inhibitor	0.00168	0.007867518
PRDX2	P32119	Peroxiredoxin-2	0.001761	0.008049537
ALB	P02768	Albumin	0.001896	0.008464298
SERPINF2	P08697	Alpha-2-antiplasmin	0.002162	0.009434925
GSN	P06396	Gelsolin	0.002225	0.009491889
