# Supplementary material for: Comparative Proteomic Profiling of Blood Plasma Revealed Marker Proteins Involved in Temporal Lobe Epilepsy
Source: Int J Mol Sci. 2024 Jul 20;25(14):7935. doi: 10.3390/ijms25147935 (PMC11276668; doi:10.3390/ijms25147935)
Supplement: Supplementary file 1 [file ijms-25-07935-s001.zip › Table S2 REVISED.pdf]

Table S2. Differently regulated proteins of MRI-positive and MRI-negative groups.

Gene ID	Protein ID	Protein name	P-value	Corrected p-value	Upregulated in group
VTN	P04004	Vitronectin	2.7E-06	0.000129912	MRI-positive
APOE	P02649	Apolipoprotein E	5.41E-06	0.000129912	MRI-positive
C8B	P07358	Complement component C8 beta chain	2.64E-05	0.000321395	MRI-positive
ACTB	P60709	Actin, cytoplasmic 1	0.000131	0.001044635	MRI-positive
A1BG	P04217	Alpha-1B-glycoprotein	0.000436	0.002770914	MRI-positive
C8A	P07357	Complement component C8 alpha chain	0.000582	0.002947387	MRI-positive
CFP	P27918	Properdin	0.000763	0.003754774	MRI-positive
NA	P01860	Immunoglobulin heavy constant gamma 3	0.001052	0.005051919	MRI-positive
PGLYRP2	Q96PD5	N-acetylmuramoyl-L-alanine amidase	0.001518	0.007107712	MRI-positive
CFH	P08603	Complement factor H	0.001576	0.007203802	MRI-positive
APOF	Q13790	Apolipoprotein F	0.001723	0.007437542	MRI-positive
A2M	P01023	Alpha-2-macroglobulin	6.8E-09	1.3053E-06	MRI-negative
PROC	P04070	Vitamin K-dependent protein C	2.46E-07	2.35788E-05	MRI-negative
CPN1	P15169	Carboxypeptidase N catalytic chain	2.81E-06	0.000129912	MRI-negative
VASN	Q6EMK4	Vasorin	4.26E-06	0.000129912	MRI-negative
F9	P00740	Coagulation factor IX	4.38E-06	0.000129912	MRI-negative
C1RL	Q9NZP8	Complement C1r subcomponent-like protein	5.29E-06	0.000129912	MRI-negative
CPN2	P22792	Carboxypeptidase N subunit 2	7.51E-06	0.00016015	MRI-negative
SERPINA3	P01011	Alpha-1-antichymotrypsin	1.28E-05	0.000241002	MRI-negative
LCAT	P04180	Phosphatidylcholine-sterol acyltransferase	1.38E-05	0.000241002	MRI-negative
PON1	P27169	Serum paraoxonase/arylesterase 1	1.56E-05	0.000248838	MRI-negative
IGFALS	P35858	Insulin-like growth factor-binding protein complex acid labile subunit	1.74E-05	0.000257294	MRI-negative
C1R	P00736	Complement C1r subcomponent	2.38E-05	0.000321395	MRI-negative
PROZ	P22891	Vitamin K-dependent protein Z	2.68E-05	0.000321395	MRI-negative
SERPING1	P05155	Plasma protease C1 inhibitor	3.22E-05	0.000353403	MRI-negative
CPB2	Q96IY4	Carboxypeptidase B2	3.31E-05	0.000353403	MRI-negative
ITIH1	P19827	Inter-alpha-trypsin inhibitor heavy chain H1	8.67E-05	0.000876142	MRI-negative
ATRN	O75882	Attractin	0.000119	0.001044635	MRI-negative
ITIH2	P19823	Inter-alpha-trypsin inhibitor heavy chain H2	0.000121	0.001044635	MRI-negative
SERPINA1	P01009	Alpha-1-antitrypsin	0.000124	0.001044635	MRI-negative
AFM	P43652	Afamin	0.000126	0.001044635	MRI-negative
F5	P12259	Coagulation factor V	0.000149	0.001146343	MRI-negative
SERPINF2	P08697	Alpha-2-antiplasmin	0.000171	0.001254726	MRI-negative
SPP2	Q13103	Secreted phosphoprotein 24	0.000176	0.001254726	MRI-negative
HPX	P02790	Hemopexin	0.000269	0.001847404	MRI-negative

SERPINC1	P01008	Antithrombin-III	0.000423	0.002770914	MRI-negative
CNDP1	Q96KN2	Beta-Ala-His dipeptidase	0.000447	0.002770914	MRI-negative
SERPIND1	P05546	Heparin cofactor 2	0.000491	0.0028647	MRI-negative
C4BPB	P20851	C4b-binding protein beta chain	0.000492	0.0028647	MRI-negative
CP	P00450	Ceruloplasmin	0.000521	0.002939335	MRI-negative
BCHE	P06276	Cholinesterase	0.000552	0.002947387	MRI-negative
CFD	P00746	Complement factor D	0.000558	0.002947387	MRI-negative
SELENOP	P49908	Selenoprotein P	0.000583	0.002947387	MRI-negative
FCN3	O75636	Ficolin-3	0.001726	0.007437542	MRI-negative
FGG	P02679	Fibrinogen gamma chain	0.001743	0.007437542	MRI-negative
CFHR4	Q92496	Complement factor H-related protein 4	0.001917	0.008003398	MRI-negative
