# Supplementary material for: Comparative Proteomic Profiling of Blood Plasma Revealed Marker Proteins Involved in Temporal Lobe Epilepsy
Source: Int J Mol Sci. 2024 Jul 20;25(14):7935. doi: 10.3390/ijms25147935 (PMC11276668; doi:10.3390/ijms25147935)
Supplement: Supplementary file 1 [file ijms-25-07935-s001.zip › Table S3 REVISED.pdf]

Table S3. Previously discovered proteins associated with epilepsy.

Protein ID	Disease / Model	Sample	Reference
A2M	Drug-resistant epilepsy	CSF	Vasil'eva, 1989
APJ/CLU	Electroshock model	Tissues	Dragunow, 1995
APJ/CLU	Kainic model	Tissues	Ma, 1992
APOA1	MTLE	Plasma	Yang, 2005
APOD	Kainic model	Tissues	Ong, 1997; Montpied, 1999
VTN	Kainic model	Tissues	Niquet, 1996
IGHG3	Idiophatic TLE	CSF	Xiao, 2009
IGHG3	Epilepsy	Serum	Haraldsson, 1992
C8B	Rolandic epilepsy	Plasma	Sun, 2020
CFP	Focal and generalized epilepsies	Plasma	Kopczynska, 2018
APOE	TLE	Plasma	Kumar, 2007
APOE	Kainic model	Tissues	Montpied, 1999
ACTB	MTLE	Tissues	Yang, 2006
C9	TLE with HS	Tissues	Aronica, 2007
EFEMP1	Lithium pilocarpine model	Tissues	Wang, 2021
EFEMP1	Drug-resistant TLE	Tissues	Koen, 2007
APOA4	Refractory epilepsy	Plasma	Saengow, 2021
AGT	TLE	Tissues	Gouveia, 2012
GSN	TLE	CSF	Peng, 2011
SELENOP	MTLE	Tissues	Yüzbaşioğlu, 2009

TLE – temporal lobe epilepsy; MTLE – mesial temporal lobe epilepsy; HS – hippocampal sclerosis; CSF – cerebrospinal fluid.
