# Supplementary material for: Comparative Proteomic Profiling of Blood Plasma Revealed Marker Proteins Involved in Temporal Lobe Epilepsy
Source: Int J Mol Sci. 2024 Jul 20;25(14):7935. doi: 10.3390/ijms25147935 (PMC11276668; doi:10.3390/ijms25147935)
Supplement: Supplementary file 1 [file ijms-25-07935-s001.zip › Table S4 REVISED.pdf]

Table S4. Association of protein expression with antiepileptic drugs medication.

Protein	Drug	Hyper/hypo expression	Reference
HPX	Phenytoin, Phenobarbital	+	Tutor, 1982
CP	Valproic acid	+	Lampón, N., 2010
BCHE	Valproic acid, Carbamazepine	-	Işık, 2015
PON1	Valproic acid, Carbamazepine	-	Işık, 2015
PON1	Gabapentin, Valproic acid, Primidone, Phenytoin, Levetiracetam	-	Beydemir, 2016
APOJ / CLU	Valproic acid	+	Nuutinen, 2010
Proteins of the coagulation system	Valproic acid	-	Krause, 2008; Cannizzaro, 2007
Proteins of the coagulation system	Topiramate	-	Frank M.C., 2009
FGG	Valproic acid	-	El-AshryR, 2020
APOA1	Valproic acid, Carbamazepine	-	Tomoum, 2008; Eirís, 2010
APOA1	Valproic acid, Carbamazepine	+	Voudris, 2006
Immunoglobulins	Phenytoin	-	Bardana, 1983; Ranua, 2005
Immunoglobulins	Carbamazepine	-	Castro, 2001; Ashrafi, 2010
BTD	Valproic acid	-	Castro-Gago, 2009; Schulpis, 2001
